# The impact of childhood trauma and cannabis use on paranoia: a structural equation model approach

**DOI:** 10.1017/S0033291725101190

**Published:** 2025-08-08

**Authors:** Giulia Trotta, Edoardo Spinazzola, Hannah Degen, Zhikun Li, Isabelle Austin-Zimmerman, Bok Man Leung, Yifei Lang, Victoria Rodriguez, Monica Aas, Lucia Sideli, Kim Wolff, Tom P. Freeman, Robin M. Murray, Chloe C. Y. Wong, Luis Alameda, Marta Di Forti

**Affiliations:** 1 Social, Genetic and Developmental Psychiatry Centre, Institute of Psychiatry, Psychology and Neuroscience, King’s College London, London, UK; 2 Department of Psychosis Studies, Institute of Psychiatry, Psychology and Neuroscience. King’s College of London, London, UK; 3 Department of Psychiatry, LKS Faculty of Medicine, The University of Hong Kong, Hong Kong SAR, China; 4 Camden Early Intervention Service, North London NHS Foundation Trust, London, UK; 5 Department of Human Science, LUMSA University, Rome, Italy; 6 King’s Forensics, Department of Analytical, Environmental & Forensic Sciences, Institute of Pharmaceutical Sciences, King’s College of London, London, UK; 7 Addiction and Mental Health Group, Department of Psychology, University of Bath, Bath, UK; 8 Service of General Psychiatry, Treatment and Early Intervention in Psychosis Program, Lausanne University Hospital (CHUV), Lausanne, Switzerland; 9 Centro Investigacion Biomedica en Red de Salud Mental (CIBERSAM), Instituto de Biomedicina de Sevilla (IBIS), Hospital Universitario Virgen del Rocio, Departamento de Psiquiatria, Universidad de Sevilla, Sevilla, Spain; 10 South London and Maudsley NHS Foundation Trust, London, UK

**Keywords:** cannabis use, childhood trauma, paranoia, standard THC unit, structural equation modeling

## Abstract

**Background:**

Childhood trauma is a well-established risk factor for psychosis, paranoia, and substance use, with cannabis being a modifiable environmental factor that exacerbates these vulnerabilities. This study examines the interplay between childhood trauma, cannabis use, and paranoia using standard tetrahydrocannabinol (THC) units as a comprehensive measure of cannabis exposure.

**Methods:**

Data were derived from the Cannabis&Me study, an observational, cross-sectional, online survey of 4,736 participants. Childhood trauma was assessed using a modified Childhood Trauma Screen Questionnaire, while paranoia was measured via the Green Paranoid Thoughts Scale. Cannabis use was quantified using weekly standard THC units. Structural equation modeling (SEM) was employed to evaluate direct and indirect pathways between trauma, cannabis use, and paranoia.

**Results:**

Childhood trauma was strongly associated with paranoia, particularly emotional, and physical abuse (*β* = 16.10, *q* < 0.001; *β* = 16.40, *q* < 0.001). Cannabis use significantly predicted paranoia (*β* = 0.009, *q* < 0.001). Interactions emerged between standard THC units and both emotional abuse (*β* = 0.011, *q* < 0.001) and household discord (*β* = 0.011, *q* < 0.001). SEM revealed a small but significant indirect effect of trauma on paranoia via cannabis use (*β* = 0.004, *p* = 0.017).

**Conclusions:**

These findings highlight childhood trauma as a primary driver of paranoia, with cannabis use amplifying its effects. While trauma had a strong direct impact, cannabis played a significant mediating role. Integrating standard THC units into psychiatric research and clinical assessments may enhance risk detection and refine intervention strategies, particularly for childhood trauma-exposed individuals.

## Introduction

Childhood trauma, including experiences such as abuse, neglect, bullying, or household discord, is a recognised risk factor for adverse mental health outcomes, such as psychosis, paranoia, and substance use (Shevlin, McAnee, Bentall, & Murphy, 2014; Varese et al., 2012; Wolitzky-Taylor et al., 2017). Among the general population, cannabis use is a modifiable environmental factor that exacerbates these vulnerabilities (Xenaki, Dimitrakopoulos, Selakovic, & Stefanis, 2023). A dose–response relationship between frequency, potency, and duration of cannabis use and the severity of paranoia is well-established (D. Freeman et al., 2008). Notably, cannabis is associated with increased paranoia, particularly in individuals with a history of trauma (D. Freeman et al., 2011).

Paranoia, a core feature of psychotic experiences characterized by excessive mistrust and suspicion, can be exacerbated by cannabis use through multiple mechanisms. It has been proposed that cannabis may contribute to paranoia by dysregulating the endocannabinoid system, which plays a crucial role in emotional regulation, stress response, and cognitive processes (Appiah-Kusi et al., 2016). Additionally, it amplifies pre-existing cognitive biases, such as maladaptive patterns of interpreting social interactions, of negative views about the self, the world, and others, which are frequently a consequence of childhood traumatic events (Alameda et al., 2020; Thoma & Daum, 2013). Research indicates that individuals with a history of childhood trauma may be especially vulnerable to the paranoia-inducing effects of cannabis, as trauma appears to heighten sensitivity to its psychoactive properties (Carlyle et al., 2021).

Childhood trauma also plays a role in shaping cannabis use patterns (Sideli et al., 2018). Exposure to early trauma increases the likelihood of cannabis initiation, particularly during adolescence and young adulthood, a critical period for brain maturation, social independence, and identity formation (Morgan et al., 2014). This bidirectional relationship suggests that childhood trauma not only increases the risk of cannabis dependence but that cannabis use, in turn, worsens trauma-related cognitive bias and social cognition deficits and emotional dysregulation (Aas et al., 2024). Indeed, recent findings from our group showed that cannabis use may mediate the pathway from childhood trauma, particularly emotional abuse and household discord, to psychosis, inducing experiences of dysphoria and paranoia that reinforce maladaptive psychological processes (Trotta et al., 2023).

Research has increasingly focused on the cumulative and interactive effects of trauma and cannabis use on psychotic symptoms, particularly paranoia, which has been shown to have a profound impairment in daily functioning, social relationships, and emotional well-being (Bebbington et al., 2013). For example, studies have shown that the combination of trauma and cannabis has synergistic effects on the development and severity of paranoia (Morgan et al., 2014). Understanding the mechanisms underlying the development of paranoia, particularly the modifiable role of cannabis and the impact of early-life trauma, can inform more effective interventions. The growing body of evidence in this area underscores the need to investigate how cannabis use moderates or mediates the relationship between early-life adversity and later mental health outcomes.

The Cannabis&Me (CAMe) study provides a unique framework to explore these complex interactions in a large, diverse, non-clinical sample of both cannabis users and non-users recruited through an online survey. We hypothesised that (1) childhood trauma exposure would be associated with more severe paranoia, following a dose–response relationship and (2) cannabis use would play both a moderating and mediating role in this association. Specifically, we predicted that individuals with greater cannabis consumption would experience heightened paranoia symptoms in response to trauma. Finally, we predicted that individuals with childhood trauma may be more likely to use cannabis, which in turn may contribute to increased paranoia symptoms.

## Methods

### Study design

The CAMe study is a cross-sectional study designed to examine the interplay between childhood trauma, cannabis use, and paranoia in a large non-clinical sample. Participants were recruited between March 2022 and July 2024 through an online survey using targeted advertisements on social media and cannabis-related forums used by adults (age ≥ 18 years). Recruitment strategies aimed to ensure diversity among cannabis users, while non-users and past users were recruited via tailored outreach to achieve a representative comparison group. Individuals with a prior diagnosis or treatment history for a psychotic disorder were excluded. All participants, when opening the link to the survey were invited to read the study information sheet and consent form. Only those who consented were able to progress with completing the survey. The study was approved by the Research Ethics Committee at King’s College London (IRAS project ID 301405) and adhered to General Data Protection Regulation (GDPR) guidelines.

### Measures

*Sociodemographic data.* Participants completed a modified version of the MRC Sociodemographic Schedule, which collected information on age, sex at birth, educational attainment, employment status, and ethnic background (Mallett, 1997).

*Childhood trauma.* Childhood trauma was assessed using a modified version of the Childhood Trauma Screen questionnaire (CTS), which was adapted for the CAMe study (Bernstein et al., 2003). This self-report evaluates exposure to multiple forms of childhood adversity (occurring between 0 and 17 years) and has been widely used in large-scale population studies and is the most used instrument in psychotic disorders (Davies et al., 2019; Davis et al., 2020; Saetren et al., 2024). The instrument includes items addressing five core domains of trauma (i.e., physical abuse, emotional abuse, sexual abuse, physical neglect, and emotional neglect), and the adapted version used for this study also includes bullying and exposure to household discord (e.g., parental conflict). The addition of these two adversities to the questionnaire was agreed upon by an expert consensus given the various reports of their association with psychosis (Morgan & Gayer-Anderson, 2016; Pastore, De Girolamo, Tafuri, Tomasicchio, & Margari, 2022). Each item required respondents to rate the frequency of their experiences on a 5-point Likert scale, ranging from ‘never true’ (1) to ‘very often true’ (5). For each type of trauma, respondents were classified as exposed if they reported moderate-to-severe experiences, corresponding to a score of 4 (‘often true’) or 5 (‘very often true’) on at least one item related to that trauma type. We therefore created seven categories of trauma subtypes (yes/no) that were used for the regression, moderation, and structural equation modeling (SEM) analyses (see below). For the descriptive analyses of the sample, participants were categorized into trauma and non-trauma according to whether they had been exposed to any of the trauma subtypes categorized following the cut-off points mentioned above.

*Cannabis use.* Cannabis use was measured using the modified Cannabis Experiences Questionnaire – (CEQ_mv_) (Di Forti et al., 2019) further updated for this study. This questionnaire captured information about cannabis use (e.g., frequency, amount, and potency). For the purpose of the analyses, we built weekly tetrahydrocannabinol (THC) units (Freeman & Lorenzetti, 2020). This measure provides a standardized approach to quantify cannabis exposure in units of THC, the primary psychoactive constituent in cannabis, which causes its mental health effects including paranoia. A key strength of the standard THC unit is that it can be applied to all cannabis products and methods of administration to give a single and direct measure of THC consumption. Participants reported their typical consumption patterns, including their frequency of use, the type used (e.g., hash, herbal, oil, and/or their specific names) and the quantity of cannabis used (e.g., g and/or ml), which were used to derive their overall consumption in standard weekly THC units. Measures of the standard THC unit are strongly correlated with cannabis use quantified by urinalysis (Petrilli et al., 2024). Using standard THC units allows for a more precise and replicable measure of cannabis use and for quantification of thresholds for harmful use. The US National Institutes of Health have endorsed the standard THC unit as a recommended measure of cannabis use and mandate investigators to report it in applicable research studies (Freeman & Lorenzetti, 2021).

*Paranoia.* Paranoia was measured using the Green Paranoid Thoughts Scale (GPTS) (Green et al., 2008). This validated scale measures two dimensions of paranoia: (1) social reference, perceptions that others are observing or commenting negatively and (2) persecutory ideation, beliefs about being targeted or harmed. Participants rated 32 items on a 5-point scale, with total scores ranging from 32 to 160.

### Statistical analysis

Statistical analyses were conducted using R studio (version 4.3.1.). Descriptive statistics summarized the sample, using means and standard deviations for continuous variables and frequencies for categorical data. Group differences were assessed using t-tests or Mann–Whitney U tests for continuous variables and chi-square tests for categorical variables. To handle missing standard THC unit data, multiple imputation was applied using the mice package in R, implementing predictive mean matching (PMM) with 20 imputations to reduce bias and maintain statistical power (Hegde et al., 2019). First, linear regression assessed associations between childhood trauma, standard THC units, and paranoia, while controlling for potential confounders, such as age, gender, years of education, and ethnicity. Second, moderation analyses were conducted to assess whether cannabis use influenced the relationship between childhood trauma and paranoia. Separate multiplicative interaction models were built for each trauma type using linear regression. Each trauma type’s effect was adjusted for standard THC unit consumption, while the standard THC unit effect was adjusted for the corresponding trauma type depending on the model, alongside sociodemographic covariates. To account for multiple comparisons, we applied false discovery rate (FDR) correction, reducing the risk of false-positive findings, with an adjusted significance threshold set at *q* < 0.05 (Benjamini & Hochberg, 1995). Third, to evaluate whether cannabis use mediated the relationship between childhood trauma and paranoia, SEM was performed (Bollen, 1989). Childhood trauma was then modeled as a latent variable, constructed from seven trauma indicators: emotional abuse, physical abuse, sexual abuse, emotional neglect, physical neglect, household discord, and bullying. These trauma types were selected based on their strong theoretical and empirical associations with paranoia, allowing the model to capture the broad impact of early adversity. The indirect effect was estimated as the product of the trauma-to-cannabis and cannabis-to-paranoia pathways, with bootstrapping (10,000 resamples) used to generate confidence intervals for robust inference. Statistical significance was set at *p* < 0.05, and effect sizes, including standardized coefficients and odds ratios, were reported to enhance interpretability. In sensitivity analyses, traditional cannabis measures (i.e., current use and frequency) were also included for comparison, and additional models were run using complete cases (non-imputed data) and stratified by gender (see Supplementary Material, Sections 4–6).

## Results

### Sample characteristics, trauma prevalence and standard THC unit exposure

Supplementary Figure S1 provides a flowchart of participants included in the study. Of the 4,858 initial online survey respondents, 122 duplicate or triplicate responses were excluded (106 duplicate and 8 triplicate), leaving 4,736 unique respondents. Among these, 1,347 participants reported never using cannabis; the remaining 3,389 lifetime cannabis users were categorized into two groups: 2,573 current cannabis users and 816 past users. Descriptions of the overall sample, as well as stratified by trauma exposure (comparing those exposed to at least one of the trauma subtypes with participants exposed to none), are shown in Table 1. Participants had an average age of 31.78 years (SD 10.46) and included slightly more males (*N* = 2632, 55.6%) than females (*N* = 2099, 44.3%) overall. However, gender distribution differed by trauma exposure, females were more represented in the trauma group (*N* = 1180, 47.5% compared to *N* = 919, 40.8% in the non-trauma group). About two-thirds of respondents identified as White or White Other (*N* = 2891, 61%), followed by British Asian (*N* = 684, 14.4%) and Black British (*N* = 623, 13.2%), with a diverse range of ethnicities represented (Supplementary Figure S2). Educational attainment averaged 16.3 years (SD = 3.9), with higher levels observed in participants not exposed to trauma. Unemployment was reported in approximately 13% of the cohort, with higher rates observed in the group exposed to trauma (*N* = 369, 15.4%). Regarding trauma exposure, approximately half of the cohort (*N* = 2482, 52%) reported experiencing some form of trauma. Among specific types of traumas, emotional and physical abuse were most frequently reported, followed by sexual abuse, neglect, exposure to household discord, and bullying. Those exposed to trauma demonstrated higher mean scores for paranoia (GPTS) compared to those without trauma exposure (54.96 versus 43.67, *p* < 0.001). The mean age at first cannabis use was 16.67 years (SD = 5.74), with no significant differences between trauma-exposed (*M* = 16.65, SD 3.81) and non-trauma participants (*M* = 16.69, SD 6.90, *p* = 0.839). Standard THC unit exposure varied widely among cannabis users. The mean total weekly standard THC unit consumption across all users was 206 (SD = 268), with a median of 112 and an interquartile range (IQR) of 232. When considering cannabis use history, past cannabis users reported significantly lower weekly THC exposure, with a mean of 82.5 (SD = 140), a median of 30, and an IQR of 94.2. In contrast, current cannabis users exhibited substantially higher weekly THC unit exposure, with a mean of 224 (SD = 283), a median of 132, and an IQR of 230.

**Table 1.** Descriptive characteristics of the sample by trauma exposure and cannabis use

	Overall	Non-trauma	Trauma	*p*-Value
*N*	4736	2254	2482	
Age (mean [SD])	31.78 (10.46)	32.11 (10.72)	31.48 (10.21)	0.039*
Sex (%)				<0.001*
Female	2099 (44.3)	919 (40.8)	1180 (47.5)	
Male	2632 (55.6)	1333 (59.1)	1299 (52.3)	
Other	5 (0.1)	2 (0.1)	3 (0.1)	
Ethnicity (%)				<0.001*
Black British	623 (13.2)	272 (12.1)	351 (14.1)	
British Asian	684 (14.4)	330 (14.6)	354 (14.3)	
Mixed	538 (11.4)	220 (9.8)	318 (12.8)	
White and White Other	2891 (61.0)	1432 (63.5)	1459 (58.8)	
Employment status = Unemployed (%)	599 (13.1)	230 (10.5)	369 (15.4)	<0.001*
Years of education (mean [SD])	16.29 (3.88)	16.70 (3.65)	15.92 (4.03)	<0.001*
Abuse = trauma (%)	1166 (24.6)		1166 (47.0)	
Neglect = trauma (%)	904 (19.1)		904 (36.4)	
Household discord = trauma (%)	1437 (30.3)		1437 (57.9)	
Bullying = trauma (%)	974 (20.6)		974 (39.2)	
GPTS (mean [SD])	49.59 (21.78)	43.67 (15.41)	54.96 (25.08)	<0.001*
Cannabis use (%)				<0.001*
Non-users	1347 (28.4)	755 (33.5)	592 (23.9)	
Past users	816 (17.2)	405 (18.0)	411 (16.6)	
Current users	2573 (54.3)	1094 (48.5)	1479 (59.6)	
Age at first use (mean [SD])	16.67 (5.74)	16.65 (3.81)	16.69 (6.90)	0.839

*Note:* The table presents the demographic, trauma, and clinical characteristics of the sample, stratified by trauma exposure (non-trauma versus trauma) and cannabis use categories (non-users, past users, current users). Statistical tests used: t-tests or ANOVA for continuous variables and chi-square tests for categorical variables. Trauma categories include abuse (emotional, physical, sexual) and neglect (emotional, physical), as well as household discord and bullying. Cannabis use refers to participants classified as current users, past users, or non-users. *Significant *p*-values (*p* < 0.05) are highlighted.

### Impact of childhood trauma subtypes and cannabis use on paranoia

**Figure 1.**
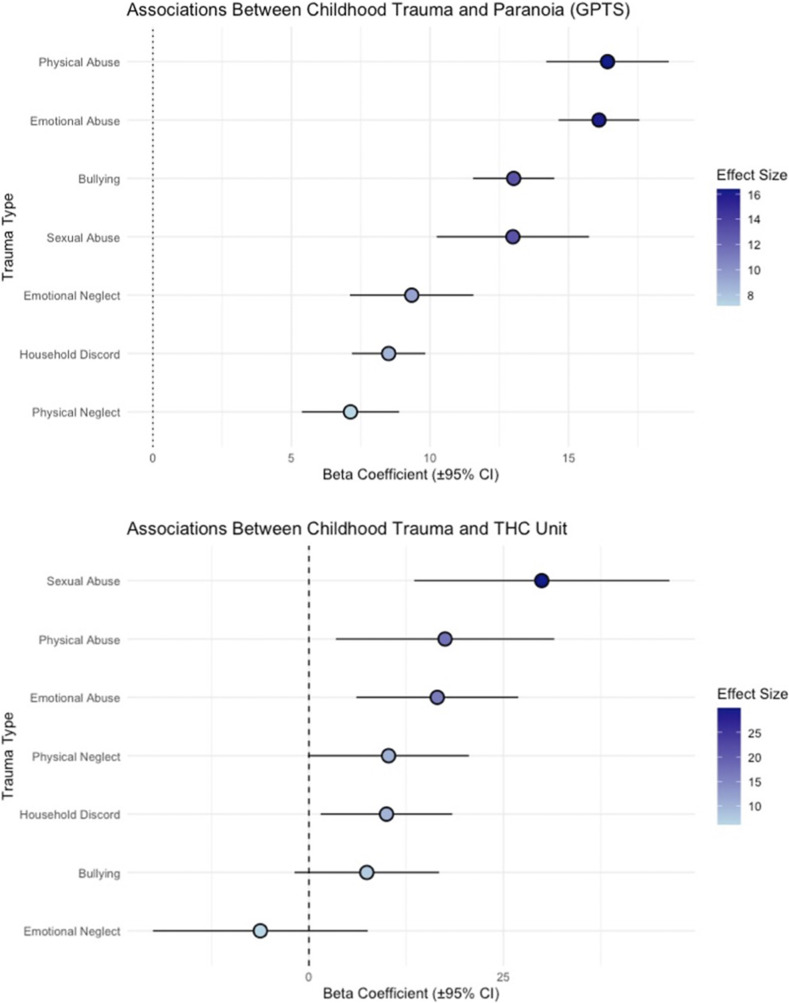
Associations between childhood trauma, paranoia (GPTS), and cannabis use (standard THC unit).

Childhood trauma was strongly associated with heightened paranoia symptoms (Figure 1). Physical abuse (*β* = 16.40, SE = 1.13, *q* < 0.001) and emotional abuse (*β* = 16.10, SE = 0.74, *q* < 0.001) emerged as the strongest predictors of paranoia. Bullying (*β* = 13.02, SE = 0.75, *q* < 0.001) and sexual abuse (*β* = 12.99, SE = 1.40, *q* < 0.001) also demonstrated significant associations with paranoia. Beyond interpersonal trauma, household discord (*β* = 8.51, SE = 0.68, *q* < 0.001) also contributed significantly to paranoia symptoms. Neglect was also significantly related to paranoia. Emotional neglect (*β* = 9.34, SE = 1.14, *q* < 0.001) and physical neglect (*β* = 7.13, SE = 0.90, *q* < 0.001) both predicted increased paranoia symptoms. In addition to trauma, higher standard THC unit exposure was significantly linked to increased paranoia symptoms (*β* = 0.009, SE = 0.002, *q* < 0.001), indicating a dose–response relationship between cannabis use and paranoia severity.

### Childhood trauma as a risk factor for cannabis use

Childhood trauma emerged as a significant predictor of standard THC unit weekly exposure (Figure 1). Sexual abuse had the strongest association, with individuals exposed to sexual abuse reporting markedly higher weekly standard THC unit consumption (*β* = 29.93, SE = 8.37, *q* = 0.002). Emotional abuse (*β* = 16.50, SE = 5.31, *q* = 0.0067) and physical abuse (*β* = 17.50, SE = 7.16, *q* = 0.037) were also positively associated with weekly standard THC unit exposure. Household discord also emerged as a significant predictor of weekly standard THC unit consumption (*β* = 9.97, SE = 4.32, *q* = 0.037). In contrast, bullying (*β* = 7.44, SE = 4.74, *q* = 0.137) and neglect showed relatively weaker effects on standard THC unit consumption; neither emotional neglect (*β* = −6.25, SE = 7.04, *q* = 0.375) nor physical neglect (*β* = 10.24, SE = 5.27, *q* = 0.073) reached strong significance thresholds, though physical neglect approached significance.

### Cannabis use as a moderator between childhood trauma and paranoia

**Figure 2.**
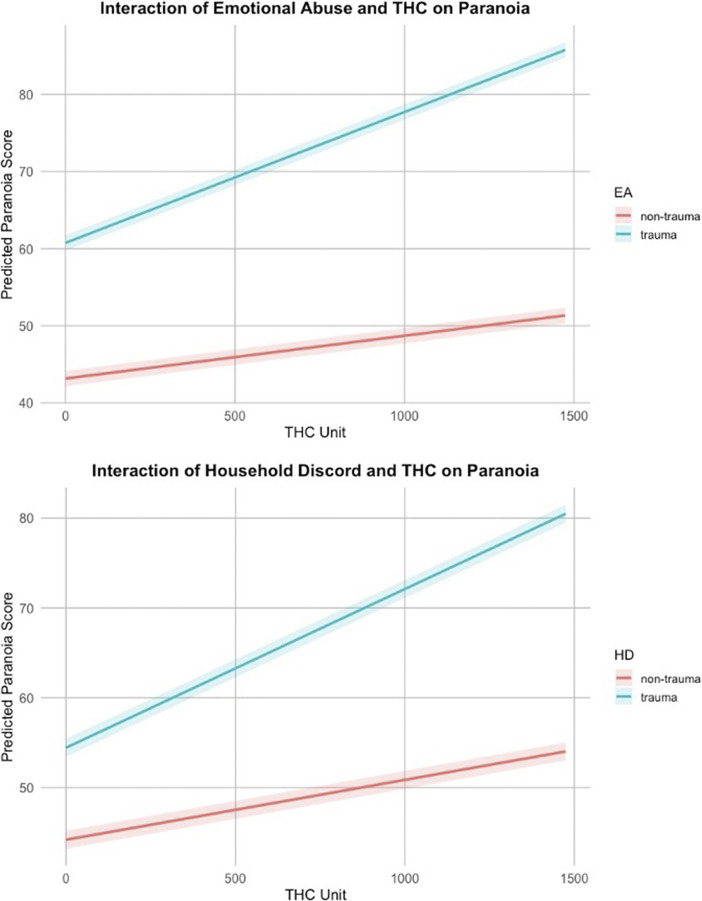
Significant interactions between childhood trauma types and cannabis use (standard THC units) on paranoia.

The moderating effect of cannabis use, as measured by standard THC units, on the relationship between different trauma types and paranoia was examined (Table 2 and Figure 2). Results indicated that emotional abuse (*β* = 0.011, SE = 0.0030, *q* = 0.00018) and household discord (*β* = 0.0086, SE = 0.0034, *q* = 0.019) significantly interacted with weekly standard THC units, amplifying paranoia symptoms. Other childhood trauma types, including bullying, physical abuse, sexual abuse, physical neglect, and emotional neglect, did not exhibit significant interaction effects with weekly standard THC units on paranoia outcomes (*q* > 0.05).

**Table 2.** Interaction effects between cannabis use (weekly standard THC units) and childhood trauma types on paranoia

Trauma type	Main effect (*β*, SE, *p*-value)	THC unit effect (*β*, SE, *p*-value)	Interaction effect (THC unit × trauma type) (*β*, SE, *p*-value)	FDR-adjusted *q*-value
Emotional neglect	9.66 (1.30), *p* < 0.001	0.0107 (0.0015), *p* < 0.001	0.0035 (0.0043), *p* = 0.419	0.419
Physical neglect	7.71 (1.02), *p* < 0.001	0.0118 (0.0016), *p* < 0.001	−0.0038 (0.0033), *p* = 0.251	0.251
Emotional abuse	14.49 (0.86), *p* < 0.001	0.0056 (0.0016), *p* < 0.001	0.0114 (0.0030), *p* < 0.001	< 0.001*
Physical abuse	16.00 (1.33), *p* < 0.001	0.0102 (0.0015), *p* < 0.001	−0.0009 (0.0041), *p* = 0.828	0.828
Sexual abuse	11.35 (1.65), *p* < 0.001	0.0095 (0.0015), *p* < 0.001	0.0085 (0.0047), *p* = 0.069	0.085
Household discord	7.02 (0.78), *p* < 0.001	0.0067 (0.0017), *p* < 0.001	0.0110 (0.0029), *p* < 0.001	< 0.001*
Bullying	12.59 (0.87), *p* < 0.001	0.0093 (0.0016), *p* < 0.001	0.0033 (0.0032), *p* = 0.299	0.299

*Note:* Covariates included in the regression models are sex, age, years of education, and ethnicity. The main effect of each trauma type is adjusted for Standard THC Unit consumption, while the THC unit effect is adjusted for the corresponding trauma type depending on the model. Interaction effects examine whether cannabis use modifies the relationship between trauma types and paranoia. *Significant associations (*p* < 0.05) are highlighted.

### Structural equation model analyses with trauma, cannabis use, and paranoia

**Figure 3.**
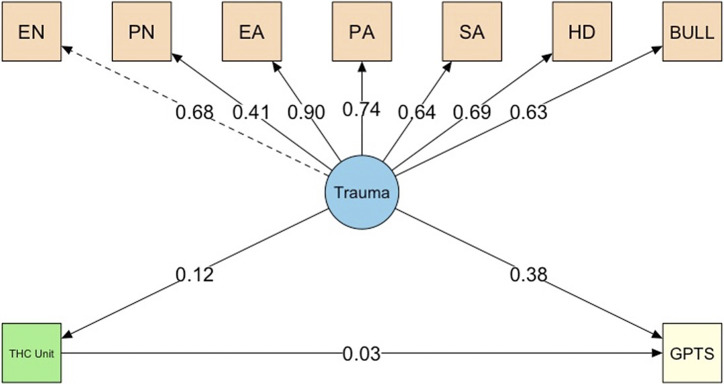
Structural equation model depicting relationships between trauma, cannabis use (standard THC units), and paranoia (GPTS).

A structural equation model (SEM) was conducted to examine the relationships between trauma exposure, weekly THC unit consumption, and paranoia (GPTS) (Figure 3). All seven indicators (i.e., emotional neglect, physical neglect, emotional abuse, physical abuse, sexual abuse, household discord, and bullying) loaded significantly onto the Trauma construct, with standardized factor loadings ranging from 0.41 (physical neglect – weaker relationship with the latent construct) to 0.90 (emotional abuse – strongest association with the latent construct), supporting the validity of the latent construct. The latent Trauma measure was directly and positively associated with weekly standard THC unit consumption (*β* = 0.12, *p* < 0.001), indicating that higher Trauma exposure is linked to increased cannabis use. In turn, standard THC unit consumption demonstrated a small but significant association with paranoia (*β* = 0.03, *p* = 0.016). Trauma had a strong and direct effect on paranoia (*β* = 0.38, *p* < 0.001) and a significant indirect (mediating) effect of Trauma on paranoia through weekly standard THC unit consumption was observed (*β* = 0.004, *p* = 0.017), though small in magnitude. The total effect of trauma on paranoia, combining both direct and indirect pathways, was *β* = 0.388, *p* < 0.001, confirming that the majority of the effect is direct rather than mediated through cannabis use. Model fit indices indicated an adequate fit to the data (CFI = 0.984, TLI = 0.978, RMSEA = 0.032, SRMR = 0.053), supporting the proposed relationships in the SEM model.

## Discussion

This is the first study to explore, in a large population sample, the interplay between childhood trauma, cannabis use, and paranoia, integrating weekly standard THC units, a comprehensive measure of cannabis use directly indexing THC consumption. The results confirm a strong association between childhood trauma and paranoia and suggest that cannabis use further exacerbates these effects in a dose-dependent and trauma-specific manner.

Furthermore, the study also corroborates the strong association between childhood trauma and paranoia, with intrusive traumas such as emotional abuse and physical abuse being the most robust predictors (Copeland et al., 2018; McKay et al., 2021; Varese et al., 2012). This is in line with prior research indicating that early emotional maltreatment disrupts self-perception, increases threat sensitivity, and heightens cognitive biases toward the self, the world, and others leading to suspicion and mistrust (Harvey, Dorahy, Vertue, & Duthie, 2012). These findings align with neurobiological models of trauma, suggesting that HPA axis dysregulation (Aas et al., 2019) and heightened amygdala (Van der Kolk, 2003) activity may underlie the increased susceptibility to paranoia in emotionally abused individuals. Additionally, bullying and sexual abuse were significantly associated with paranoia, underscoring the role of peer and interpersonal victimization in shaping paranoid thinking. Beyond direct abuse, household discord was also a significant predictor of paranoia, suggesting that exposure to family conflict may heighten threat sensitivity. Neglect, though less strongly associated, still contributed to paranoia, reflecting the impact of early emotional deprivation on mistrust and hypervigilance (Alameda et al., 2021; Bailey et al., 2018; Humphrey, Bucci, Varese, Degnan, & Berry, 2021).

Beyond trauma, higher standard weekly THC unit exposure was significantly linked to paranoia, indicating a dose–response relationship between cannabis use and paranoia severity. Unlike traditional cannabis measures (e.g., current use and frequency), standard THC units provide a more granular understanding of cannabis exposure and show with greater precision the previously reported dose-dependent relationship between cannabis and paranoia (Freeman & Lorenzetti, 2020). While the dose-dependent effect of THC on paranoia was smaller in magnitude compared to trauma, it remained statistically significant, indicating that cannabis plays a role in exacerbating paranoia even after accounting for direct effects of trauma on paranoia.

Our results suggest that cannabis use amplifies the paranoia-inducing effects of trauma, but this effect is trauma-type specific. Emotional abuse and household discord significantly interacted with weekly standard THC units, demonstrating that individuals with these specific trauma histories are particularly vulnerable to cannabis-induced paranoia. These findings align with cognitive theories that early emotional maltreatment increases susceptibility to maladaptive interpretation of social cues, making individuals more prone to paranoia-enhancing effects of cannabis (Luke & Banerjee, 2013 *;* McCrory, Ogle, Gerin, & Viding, 2019). Additionally, previous studies have shown that cannabinoid-induced dysregulation of the endocannabinoid system may be more pronounced in trauma-exposed individuals (Bielawski, Albrechet-Souza, & Frydecka, 2021). Conversely, the lack of significant interactions for other trauma types suggests that cannabis use does not uniformly moderate the trauma–paranoia relationship but instead exercises differential effects based on trauma type. Moreover, additional analyses stratified by gender suggested that, while trauma emerged as a robust predictor of paranoia across genders, the specific trauma subtypes and interaction patterns differed across genders. Males showed broader trauma sensitivity, whereas females demonstrated heightened reactivity to cannabis. The SEM analysis provided an integrated framework for understanding the relationship between trauma, weekly standard THC unit exposure, and paranoia. By modeling trauma as a latent construct, we captured its multidimensional impact on paranoia. Trauma was directly linked to paranoia, with THC unit consumption partially mediating this relationship, suggesting that cannabis use acts as a mediator of paranoia symptoms rather than a sole explanatory factor. The indirect effect of trauma on paranoia via THC use was small but statistically significant, indicating that individuals with high trauma exposure may be more likely to experience paranoia due to their cannabis use. These findings highlight the need to consider both early-life trauma and substance use behaviors in clinical assessments of paranoia risk.

This study has several strengths. First, the use of a large, diverse non-clinical community-based urban sample enhances the generalizability of the findings; second, the novelty of including weekly standard THC units as a comprehensive and direct measure of THC consumption. Third, the application of SEM allowed for a rigorous examination of the direct and indirect pathways linking trauma, cannabis use, and paranoia. However, some limitations should be acknowledged. The cross-sectional design precludes causal inferences, and reliance on self-reported trauma and cannabis use introduces potential biases, such as recall errors and social desirability effects (Danese & Widom, 2021). In particular, retrospective evaluation of childhood trauma may be subject to underreporting, especially for sensitive experiences such as sexual abuse, due to stigma, memory repression, recall bias, or discomfort disclosing such information (Hardt & Rutter, 2004). This may lead to conservative estimates of the association between certain types of trauma and paranoia. We used dichotomous trauma thresholds to ensure clinical relevance, continuous models using the raw total score were explored but showed poorer fit and interpretability in line with previous work from our group (Alameda et al., 2023). Moreover, the non-trauma group should not be considered as individuals with zero trauma exposure but rather as those with none or only mild forms of trauma. Additionally, sensitivity analyses using only participants with non-missing (i.e., non-imputed) THC unit data showed a small, non-significant indirect effect of trauma via cannabis on paranoia, which did not replicate the significant positive effect observed in the imputed model. This discrepancy likely reflects reduced power and altered THC distribution due to the exclusion of participants with missing data. Nevertheless, in a subsample of the cannabis users, who also completed a face-to-face assessment, weekly THC units were highly correlated with THC blood levels (see Supplementary Material, Section 3). Moreover, while online surveys facilitate large-scale data collection, they may underrepresent certain populations, particularly individuals with limited digital access, thereby introducing sampling bias (Johnson, Adams, & Byrne, 2024). Additionally, while we controlled for key covariates such as age, sex, education, and ethnicity, unmeasured confounders may still influence the observed associations, such as the strength of cannabis (THC content) and/or route of administration. Future research should adopt longitudinal designs to establish causal relationships and explore neurobiological mechanisms underlying the interplay between trauma, cannabis use, and paranoia.

These findings have implications for both clinical practice and public health. First, given the amplifying effects of cannabis use on paranoia among individuals who have experienced emotional abuse and household discord, cannabis use harm reduction strategies should be implemented. These could include (i) psychoeducation about the impact of heavy cannabis use on paranoia risk in trauma-exposed individuals; (ii) introduction of weekly THC unit thresholds to guide safer cannabis use; and (iii) alternative coping strategies for managing stress and anxiety. Second, the strong association between childhood trauma and paranoia highlights the importance of early screening for trauma exposure in individuals presenting with paranoid thinking, particularly those who use cannabis regularly. Screening for emotional abuse, household discord, and early cannabis exposure could help identify high-risk individuals who may benefit from targeted psychological intervention. Additionally, interventions such as cognitive-behavioral therapy and eye movement desensitisation and reprocessing may be beneficial in addressing both trauma-related distress and maladaptive coping strategies such as heavy cannabis use (Buhmann et al., 2024; Perez-Dandieu & Tapia, 2014). Finally, the findings support the need for public health policies and campaigns that inform the population, particularly trauma-exposed individuals, about the risk of cannabis consumption.

## Conclusions

This study underscores the complex interplay between childhood trauma, cannabis exposure, and paranoia, demonstrating that trauma is a strong predictor of paranoia, with cannabis use further exacerbating this liability. The findings highlight the need for trauma-informed clinical approaches and cannabis use harm reduction strategies to mitigate the psychological risks associated with high levels of cannabis use, particularly in trauma-exposed individuals. Future research should focus on identifying weekly standard THC unit thresholds of harmful use, employing longitudinal designs to establish causality, and identifying the biological and psychological mechanisms underlying these associations.

Additionally, intervention strategies should aim at reducing paranoia symptoms in individuals with a history of trauma, integrating early trauma screening, targeted psychological interventions, and cannabis harm-reduction programs, with weekly standard THC unit thresholds for harm modelled similarly to alcohol units (Marlatt & Witkiewitz, 2002). By addressing both trauma and cannabis use simultaneously, it may be possible to develop more effective and personalized interventions to improve mental health outcomes and reduce the burden of paranoia in vulnerable populations.

## Supporting information

Trotta et al. supplementary material Trotta et al. supplementary material

## Supplementary material

The supplementary material for this article can be found at http://doi.org/10.1017/S0033291725101190.
