# Supplementary material for: The impact of childhood trauma and cannabis use on paranoia: a structural equation model approach
Source: Psychol Med. 2025 Aug 8;55:e220. doi: 10.1017/S0033291725101190 (PMC12360688; doi:10.1017/S0033291725101190)
Supplement: Trotta et al. supplementary material [file S0033291725101190sup001.docx]

**The impact of Childhood Trauma and Cannabis Use on Paranoia: A Structural Equation Model Approach – Online Supplementary Material**

Giulia Trotta^1^, Edoardo Spinazzola^2^, Hannah Degen^2^, Zhikun Li^1^, Isabelle Austin-Zimmerman^1^, Bok Man Leung^2,3^, Yifei Lang^2^, Victoria Rodriguez^2,4^, Monica Aas^1^, Lucia Sideli^5^, Kim Wolff^6^, Tom P. Freeman^7^, Robin M. Murray^2^, Chloe C. Y. Wong^1^, Luis Alameda^2,8,9*^, and Marta Di Forti^1,10*^

^1^ Social, Genetic and Developmental Psychiatry Centre, Institute of Psychiatry, Psychology and Neuroscience, King’s College London, London, UK.

^2^ Department of Psychosis Studies, Institute of Psychiatry, Psychology and Neuroscience. King’s College of London, London, UK.

^3^ Department of Psychiatry, LKS Faculty of Medicine, The University of Hong Kong, Hong Kong SAR, China.

^4^ Camden Early Intervention Service, North London NHS Foundation Trust. London, UK.

^5^ Department of Human Science, LUMSA University, Rome, Italy.

^6^ King’s Forensics, Department of Analytical, Environmental & Forensic Sciences, Institute of Pharmaceutical Sciences, King’s College of London, London, UK.

^7^ Addiction and Mental Health Group, Department of Psychology, University of Bath, UK.

^8^ Service of General Psychiatry, Treatment and Early Intervention in Psychosis Program, Lausanne. University Hospital (CHUV), Lausanne, Switzerland

^9^ Centro Investigacion Biomedica en Red de Salud Mental (CIBERSAM); Instituto de Biomedicina de Sevilla (IBIS), Hospital Universitario Virgen del Rocio, Departamento de Psiquiatria, Universidad de Sevilla, Sevilla, Spain.

^10^ South London and Maudsley NHS Foundation Trust, London

^*^Both authors contributed equally

[1. Participant Recruitment, Sample Composition, and Subgroup Breakdown 4](#_Toc200722075)

[1.1. Recruitment and Sample Composition 4](#_Toc200722076)

[Figure S1. Flowchart of participants included in the study. 4](#_Toc200722077)

[1.2. Ethnic Composition of the Sample and Comparison with General Population 5](#_Toc200722078)

[Figure S2. Ethnic Composition: London (ONS 2021) vs. CAMe Survey. 6](#_Toc200722079)

[1.3. Demographic and Clinical Characteristics by Cannabis Use 7](#_Toc200722080)

[***Table S1.*** *Descriptive Characteristics of the Sample by Cannabis Use* 8](#_Toc200722081)

[1.4. Paranoia Scores by Trauma Subtype and Exposure Status 9](#_Toc200722082)

[***Table S2.*** *Paranoia Scores (GPTS) by Trauma Type and Exposure Status* 9](#_Toc200722083)

[2. Collinearity Assessment Among Trauma Variables 10](#_Toc200722084)

[Figure S3. Heatmap of Correlations Among Trauma Variables. 11](#_Toc200722085)

[3. THC Unit as a Measure of Cannabis Consumption 12](#_Toc200722086)

[3.1. Methodological Details on THC Unit Definition and Handling Missingness 12](#_Toc200722087)

[3.2. THC Unit Validation Against Objective THC Blood Levels 13](#_Toc200722088)

[3.3. Dose-Dependent Relationship Between THC Unit and Paranoia 13](#_Toc200722089)

[Figure S4. Dose-dependent Relationship Between THC Unit and Paranoia. 14](#_Toc200722090)

[4. Sensitivity analyses with traditional cannabis measures 15](#_Toc200722091)

[4.1. Cannabis use and its association with Paranoia 15](#_Toc200722092)

[4.2. Childhood Trauma as a determinant of Cannabis Use 15](#_Toc200722093)

[4.3. Cannabis Use as a Moderator Between Childhood Trauma and Paranoia 16](#_Toc200722094)

[4.4. Structural Equation Model Insights on Trauma, Cannabis Use, and Paranoia 16](#_Toc200722095)

[Figure S5. Structural Equation Model – Traditional Cannabis Measures. 17](#_Toc200722096)

[4.5. Comparing THC Unit Analyses with Traditional Cannabis Use Measures 18](#_Toc200722097)

[5. Stratified Analyses by Gender 19](#_Toc200722098)

[Figure S6A. Structural Equation Model – Males. 20](#_Toc200722099)

[Figure S6B. Structural Equation Model – Females. 21](#_Toc200722100)

[6. Sensitivity Analyses Using Complete Cases 22](#_Toc200722101)

[Figure S7. Structural Equation Model – Complete Cases. 22](#_Toc200722102)

[7. Clinical Thresholds of Paranoia 23](#_Toc200722103)

[7.1. Defining Paranoia Severity Categories 23](#_Toc200722104)

[Figure S8. Density plot of GPTS total scores with Clinical Thresholds. 24](#_Toc200722105)

[7.2. Paranoia Severity Across Trauma Exposure 25](#_Toc200722106)

[Figure S9. Proportion of Paranoia Severity by Trauma Exposure. 25](#_Toc200722107)

[7.3. THC Unit Distribution by Paranoia Category 26](#_Toc200722108)

[Figure S10. THC Unit Distribution Across Paranoia Categories. 26](#_Toc200722109)

[7.4. THC by Paranoia and Trauma Exposure 27](#_Toc200722110)

[Figure S11. THC Unit Consumption by Paranoia Levels and Trauma Exposure. 28](#_Toc200722111)

[7.5. Bridging General Population Findings to Clinical Relevance 28](#_Toc200722112)

1. Participant Recruitment, Sample Composition, and Subgroup Breakdown

## *1.1. Recruitment and Sample Composition*

As part of the Cannabis&Me (CAMe) study, 4,858 participants were recruited through an online survey. After excluding 106 duplicate and 8 triplicate responses, a total of 4,736 unique participants remained in the final sample (**Figure S1**). Among them, 1,347 participants (28.5%) reported never using cannabis, while the remaining 3,389 individuals (71.5%) were classified as lifetime cannabis users. This group was further divided into current cannabis users (n = 2,573, 76.0%) and past users (n = 816, 24.0%), with past users defined as those who had stopped using cannabis more than a year prior to the survey.

*
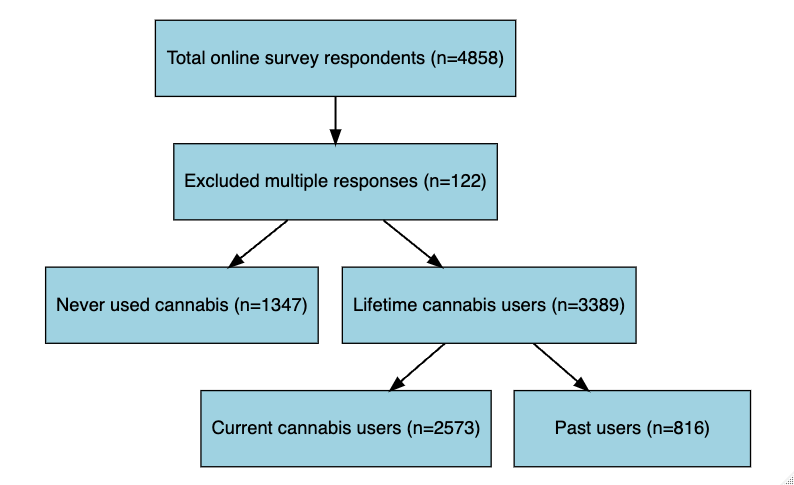
*

Figure S1. Flowchart of participants included in the study.

A total of 4,858 responses were collected through the online survey. After removing 122 duplicate or triplicate responses (106 duplicate and 8 triplicate), 4,736 unique responses remained. All respondents, including never users of cannabis, were retained for analysis. Lifetime cannabis users (n = 3,389) were further categorised into current cannabis users (n = 2,573) and past cannabis users (n = 816).

## *1.2. Ethnic Composition of the Sample and Comparison with General Population*

Comparing the ethnic distribution of the CAMe survey sample with the 2021 Office for National Statistics (ONS) estimates for London, **Figure S2** highlights some differences in ethnic representation. Overall, our sample reflects the diversity of the London population; however, White British participants were overrepresented compared to the general population, while other ethnic groups, such as British Asian and some Mixed heritage groups, were slightly underrepresented. These differences were accounted for in our analyses, as all regressions were adjusted for ethnicity.


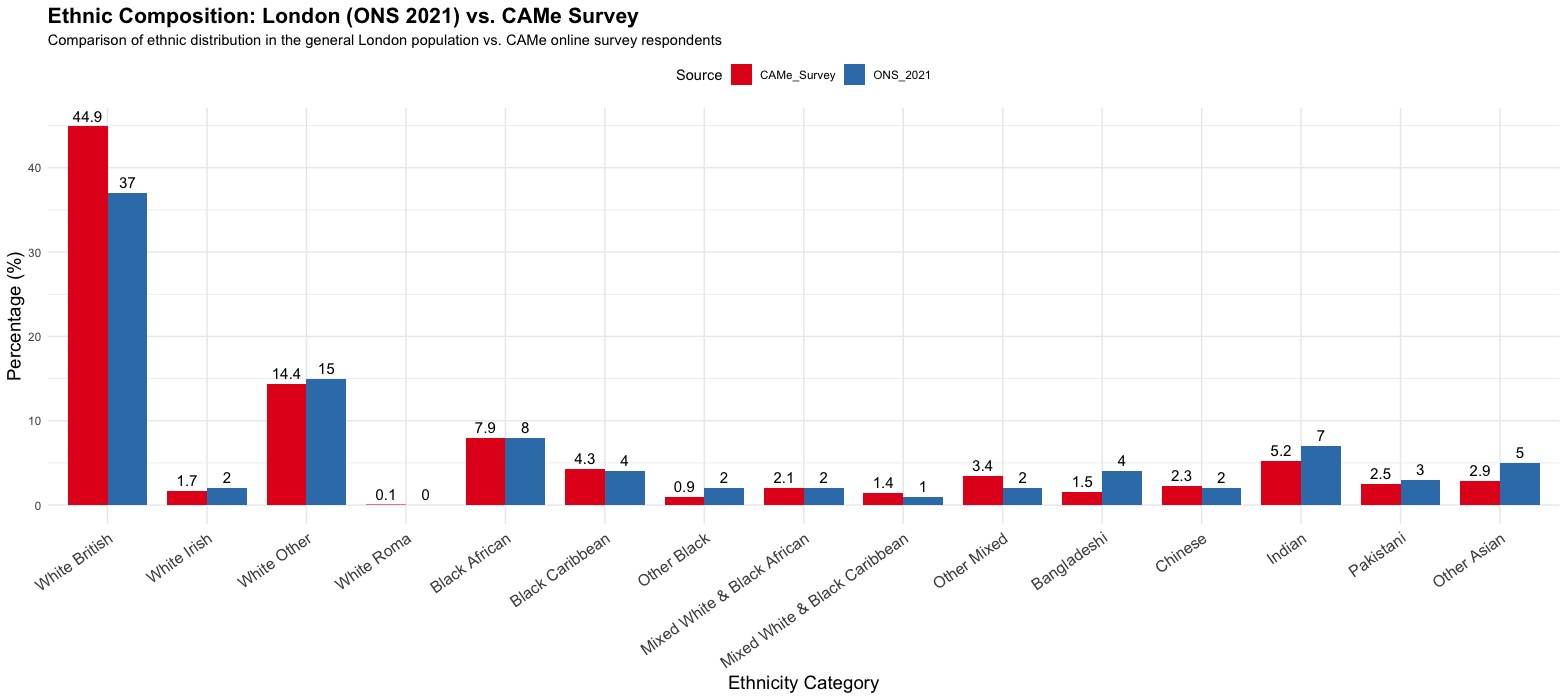


Figure S2. Ethnic Composition: London (ONS 2021) vs. CAMe Survey.

This figure compares the ethnic distribution of the CAMe survey sample and the general London population (ONS 2021 estimates). The x-axis represents different ethnic groups, while the y-axis displays the percentage of individuals within each category. Red bars represent the CAMe survey sample, while blue bars represent the ONS 2021 London population data. The figure highlights small differences in representation between the two samples, particularly the overrepresentation of White British participants and some Black ethnic groups in the CAMe survey compared to the general London population.

## *1.3. Demographic and Clinical Characteristics by Cannabis Use*

**Table S1** presents the demographic, trauma exposure, and clinical characteristics of the sample, categorised by cannabis use status (i.e., non-users, past users, and current users). The mean age of the sample was 31.78 years (SD = 10.46), with current users being younger on average (29.73 years) compared to past users (34.68 years) and never users (33.93 years). The majority of participants were male (55.6%), particularly among current cannabis users (66.4%), in contrast, non-users were predominantly female (60.7%). Unemployment was more common among current cannabis users (16.1%) compared to non-users (9.3%). Similarly years of education were slightly lower in cannabis users compared to the other groups. Trauma exposure, across all categories (i.e., abuse, neglect, household discord, and bullying), was significantly higher in cannabis users, especially current users. The mean paranoia score (GPTS total score) was also significantly higher for current cannabis users (M = 51.35, SD = 22.44) compared to non-users (M = 46.67, SD = 19.63).

### ***Table S1.*** *Descriptive Characteristics of the Sample by Cannabis Use*

|  | Overall | Non-users | Past users | Current users | p-Value |
| --- | --- | --- | --- | --- | --- |
| n | 4736 | 1347 | 816 | 2573 |  |
| Age (mean (SD)) | 31.78 (10.46) | 33.93 (11.69) | 34.68 (10.79) | 29.73 (9.15) | <0.001 |
| Sex (%) |  |  |  |  | <0.001 |
| Female | 2099 (44.3) | 817 (60.7) | 419 (51.3) | 863 (33.5) |  |
| Male | 2632 (55.6) | 529 (39.3) | 395 (48.4) | 1708 (66.4) |  |
| Other | 5 (0.1) | 1 (0.1) | 2 (0.2) | 2 (0.1) |  |
| Ethnicity (%) |  |  |  |  | <0.001 |
| Black British | 623 (13.2) | 243 (18.0) | 97 (11.9) | 283 (11.0) |  |
| British Asian | 684 (14.4) | 308 (22.9) | 88 (10.8) | 288 (11.2) |  |
| Mixed | 538 (11.4) | 111 (8.2) | 78 (9.6) | 349 (13.6) |  |
| White and White Other | 2891 (61.0) | 685 (50.9) | 553 (67.8) | 1653 (64.2) |  |
| Employment status = Unemployed (%) | 599 (13.1) | 120 (9.3) | 78 (9.9) | 401 (16.1) | <0.001 |
| Years of Education (mean (SD)) | 16.29 (3.88) | 17.18 (3.62) | 17.24 (3.82) | 15.53 (3.86) | <0.001 |
| Abuse = trauma (%) | 1166 (24.6) | 244 (18.1) | 189 (23.2) | 733 (28.5) | <0.001 |
| Neglect = trauma (%) | 904 (19.1) | 215 (16.0) | 152 (18.6) | 537 (20.9) | 0.001 |
| Household Discord = trauma (%) | 1437 (30.3) | 300 (22.3) | 240 (29.4) | 897 (34.9) | <0.001 |
| Bullying = trauma (%) | 974 (20.6) | 185 (13.7) | 174 (21.3) | 615 (23.9) | <0.001 |
| GPTS (mean (SD)) | 49.59 (21.78) | 46.67 (19.63) | 48.86 (22.50) | 51.35 (22.44) | <0.001 |

The table presents the demographic, trauma, and clinical characteristics of the sample, stratified by cannabis use categories (Non-users, Past users, Current users). Statistical tests used: t-tests or ANOVA for continuous variables and chi-square tests for categorical variables. Trauma categories include abuse (i.e., emotional, physical, sexual) and neglect (i.e., emotional, physical), as well as household discord and bullying. Significant p-values (p < 0.05) are highlighted.

## *1.4. Paranoia Scores by Trauma Subtype and Exposure Status*

**Table S2** provides a detailed summary of GPTS scores stratified by trauma subtype. This breakdown improves clarity on how paranoia scores vary by specific childhood trauma. The table demonstrates a consistent trend of higher paranoia scores among participants reporting moderate-to-severe trauma across all subtypes.

### ***Table S2.*** *Paranoia Scores (GPTS) by Trauma Type and Exposure Status*

| Trauma Type | Trauma Status | N | GPTS (mean (SD)) |
| --- | --- | --- | --- |
| EN | Non-trauma  Trauma | 4350  386 | 48.7 (21.0)  59.4 (27.3) |
| PN | Non-trauma  Trauma | 4054  682 | 48.4 (20.5)  56.6 (27.2) |
| EA | Non-trauma  Trauma | 3767  969 | 46.1 (18.1)  63.1 (28.6) |
| PA | Non-trauma  Trauma | 4354  382 | 48.2 (20.3)  65.2 (30.2) |
| SA | Non-trauma  Trauma | 4483  253 | 48.8 (21.0)  63.4 (29.3) |
| HD | Non-trauma  Trauma | 3299  1437 | 46.7 (19.4)  56.2 (25.2) |
| BULL | Non-trauma  Trauma | 3762  974 | 46.8 (18.8)  60.3 (28.2) |

EN: Emotional Neglect; PN: Physical Neglect; EA: Emotional Abuse; PA: Physical Abuse; SA: Sexual Abuse; HD: Household Discord; BULL: Bullying; GPTS: Green Paranoia Trait Scale.

2. Collinearity Assessment Among Trauma Variables

A collinearity assessment was conducted for the trauma variables included in the analyses by calculating a correlation matrix and Variance Inflation Factors (VIFs). **Figure S3** presents the correlation matrix of the trauma variables (i.e., emotional neglect [EN], physical neglect [PN], emotional abuse [EA], physical abuse [PA], sexual abuse [SA], household discord [HD], and bullying [BULL]), showing low-to-moderate correlations. Emotional abuse and household discord exhibited the highest correlation (r = 0.41), followed by emotional abuse and bullying (r = 0.34). These findings indicate that while some relationships exist between trauma variables, there is no excessive overlap or redundancy among the predictors. This was further confirmed by the VIFs, with all values ranging from 1.09 to 1.48—well below the commonly accepted threshold of 5—indicating that multicollinearity is not a concern in this model. Thus, the trauma variables independently contribute to the model without inflating standard errors. Overall, these results suggest that while some interrelations exist among the trauma variables, collinearity is minimal and does not affect the interpretability or robustness of the analyses.


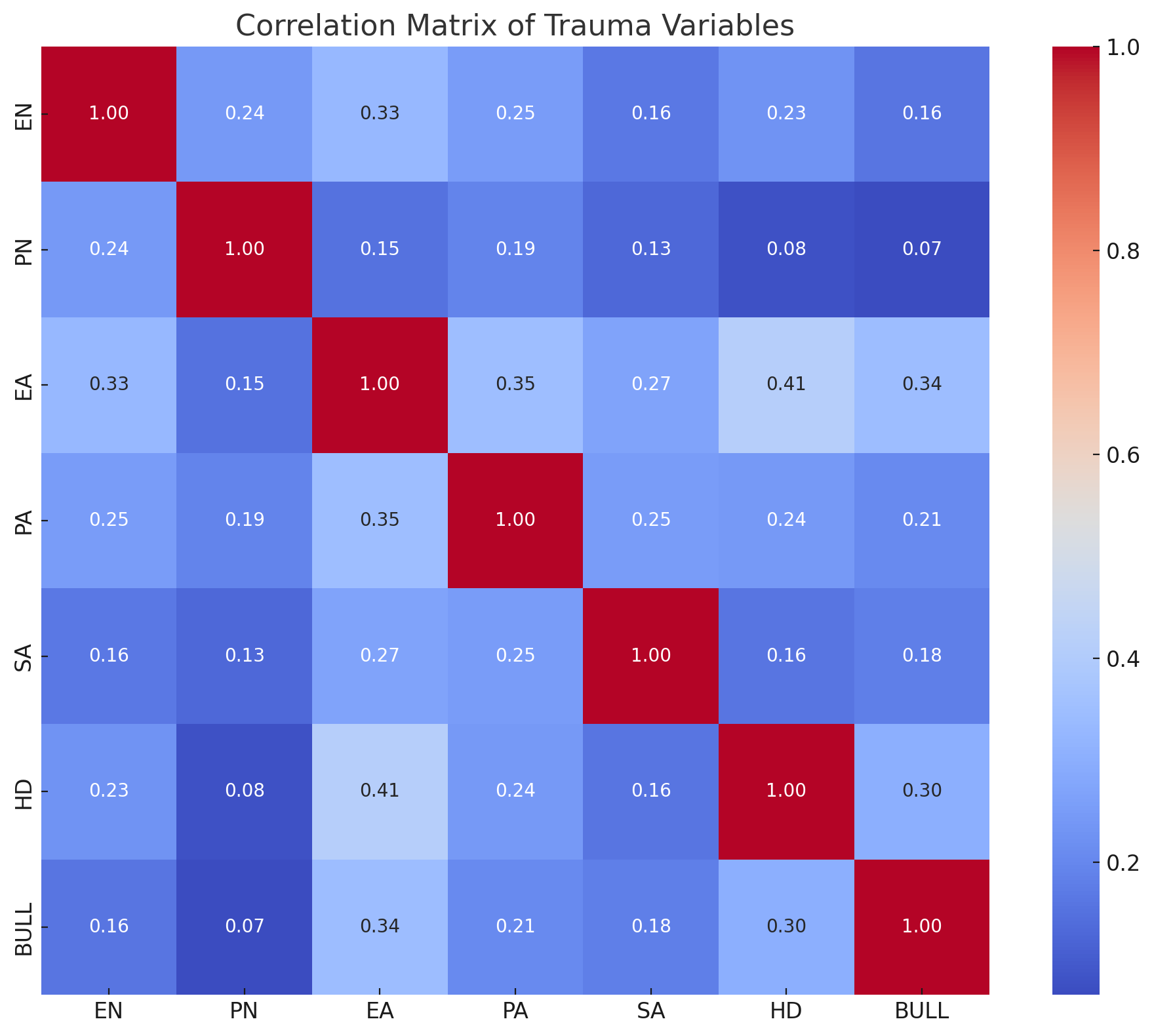


Figure S3. Heatmap of Correlations Among Trauma Variables.

This heathmap displays the correlations among seven trauma variables: Emotional Neglect (EN), Physical Neglect (PN), Emotional Abuse (EA), Physical Abuse (PA), Sexual Abuse (SA), Household Discord (HD), and Bullying (BULL). The colour gradient represents the magnitude and direction of the correlations, with darker red indicating stronger positive correlations (closer to 1) and darker blue representing weaker or no correlation (closer to 0).

3. THC Unit as a Measure of Cannabis Consumption

3.1. Methodological Details on THC Unit Definition and Handling Missingness

Both the quantity and potency of tetrahydrocannabinol (THC), the primary psychoactive component of cannabis, are incorporated into the THC unit—a standardised and more precise measure of cannabis consumption. THC units are calculated as the dose of THC per use occasion, providing a more robust and objective metric for cannabis use.

In this study, THC unit values were derived from self-reported questionnaires, with a value of 0 assigned to non-users. Among lifetime cannabis users (n = 3,389), THC unit data were missing for 846 individuals, representing 25.1% of this group. When analysing current and past users separately, missingness rates differed: current cannabis users (n = 2,573) had a lower missingness rate, with 304 observations missing THC unit data (11.89%), whereas past cannabis users had THC unit data missing for 542 individuals, representing a missingness rate of 66.42%. The higher reporting accuracy among current cannabis users likely reflects better recall of recent consumption patterns compared to past users.

To address missing THC unit data, multiple imputation by chained equations (MICE) was performed. The imputation included key cannabis-related variables (e.g., THC unit, current cannabis use, and frequency of cannabis use), sociodemographics (e.g., age, sex, ethnicity, employment status, years of education), trauma variables (e.g., abuse, neglect, household discord, bullying), and clinical outcomes (e.g., paranoia). Predictive mean matching (PMM) was applied for continuous variables, while logistic regression and multinomial logistic regression were used for binary and categorical variables, respectively.

Five imputed datasets were generated, and analyses were pooled to account for variability across imputations, ensuring robust and unbiased estimates. This approach mitigated potential biases associated with missing data while preserving the accuracy and interpretability of the analyses.

## *3.2. THC Unit Validation Against Objective THC Blood Levels*

To assess the validity of the THC unit as a measure of cannabis consumption, we compared self-reported THC unit estimates and cannabis use frequency with objective THC blood levels in a subsample of participants who attended a face-to-face assessment. THC blood concentration (M = 11.5 ng/mL, SD = 10.8) was significantly correlated with self-reported THC unit estimates (r = 0.77, p < 0.001), indicating a strong association between the THC unit and objective cannabis exposure. Similarly, frequency of cannabis use also showed a significant, though slightly lower, correlation with THC blood levels (r = 0.63, p < 0.001). These findings suggest that the THC unit provides a more precise estimate of cannabis exposure than traditional self-reported frequency measures. Given its stronger correlation with biological THC levels, the THC unit captures both the potency and quantity of cannabis consumed, making it a superior measure for assessing cannabis exposure in research settings.

## *3.3. Dose-Dependent Relationship Between THC Unit and Paranoia*

To illustrate the dose-dependent relationship between THC use and paranoia, we generated a scatter plot depicting the association between THC unit consumption and total paranoia scores (**Figure S4**). The scatter plot shows a positive association between THC use and paranoia scores, with higher THC consumption associated with greater paranoia severity. A LOESS smoothing line was applied to capture potential non-linear trends, which suggests that while the association remains relatively weak at lower THC levels, paranoia scores tend to increase more consistently at higher THC unit consumption. This visualisation demonstrates how THC use as a continuous measure provides greater precision compared to categorical cannabis use frequency.


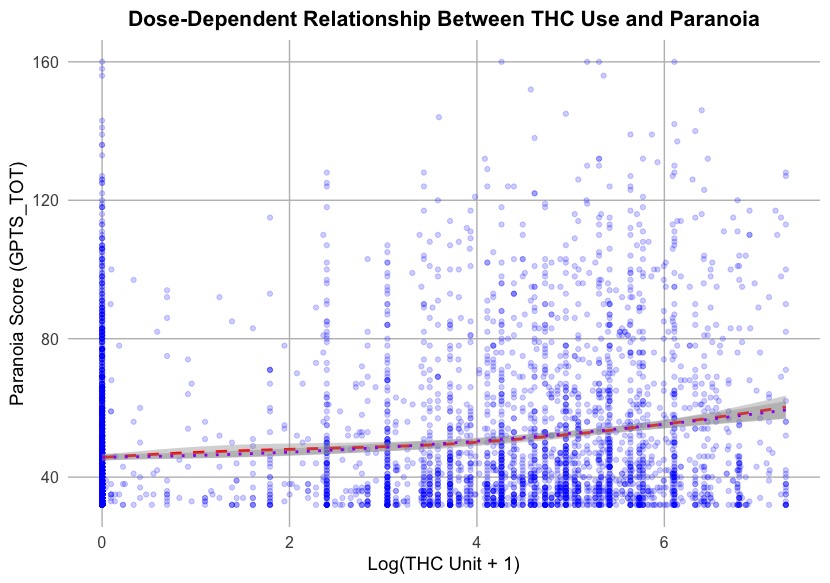


Figure S4. Dose-dependent Relationship Between THC Unit and Paranoia.

This scatter plot visualises the relationship between THC unit consumption (log-transformed) and paranoia severity (GPTS total score). Each blue dot represents an individual participant, with increased THC consumption generally associated with higher paranoia scores. The red dashed line represents a LOESS smoothing curve, capturing non-linear patterns in the data. The purple dotted line represents a polynomial regression model (quadratic fit), providing an additional perspective on the relationship between THC unit and paranoia severity. Given the highly skewed distribution of THC units, we applied a log transformation (log(THC Unit + 1)) to improve interpretability and reduce the impact of extreme values.

4. Sensitivity analyses with traditional cannabis measures

4.1. Cannabis use and its association with Paranoia

Paranoia scores were higher among current cannabis users compared to individuals who had never used cannabis (β = 6.24, SE = 0.92, p < 0.001). Furthermore, a dose-response relationship was observed, with individuals using cannabis more than once a week showing elevated paranoia scores compared to infrequent users (β = 8.36, SE = 1.01, p < 0.001).

4.2. Childhood Trauma as a determinant of Cannabis Use

Childhood trauma emerged as a significant determinant of cannabis use patterns, particularly current cannabis use and its frequency. Individuals exposed to emotional abuse during childhood were significantly more likely to report current cannabis use compared to those without such experiences (OR = 2.35, SE = 0.34, p < 0.001). Similarly, physical abuse was strongly associated with an increased risk of current cannabis use (OR = 1.89, SE = 0.29, p < 0.001). Bullying during childhood also predicted current cannabis use, with individuals reporting bullying experiences showing higher odds of cannabis use (OR = 1.72, SE = 0.26, p = 0.002). While neglect was a significant factor, its effects were relatively smaller than those associated with abuse-related traumas (OR = 1.48, SE = 0.21, p = 0.01). Notably, individuals exposed to household discord were more likely to engage in frequent cannabis use (OR = 1.94, SE = 0.31, p < 0.001). Among current cannabis users, trauma exposure also influenced frequency of use. For example, individuals who experienced sexual abuse during childhood had a significantly elevated risk of frequent cannabis use (OR = 2.12, SE = 0.37, p < 0.001). Similarly, emotional neglect was associated with a higher frequency of cannabis use among current users (OR = 1.65, SE = 0.28, p = 0.003).

4.3. Cannabis Use as a Moderator Between Childhood Trauma and Paranoia

Cannabis use significantly moderated the relationship between childhood trauma and paranoia. For example, individuals who experienced bullying and were also current cannabis users showed significantly higher paranoia scores compared to non-users (interaction term β = 2.47, SE = 0.89, p = 0.004). Similarly, frequent cannabis users with a history of emotional abuse exhibited exacerbated paranoia (interaction term β = 3.85, SE = 1.12, p < 0.001).

4.4. Structural Equation Model Insights on Trauma, Cannabis Use, and Paranoia

The SEM model (**Figure S5**) confirmed that childhood trauma had a strong direct effect on paranoia (β = 0.38, p < 0.001), reinforcing its role as a key predictor of paranoid thinking. Trauma was also associated with increased cannabis use (β = 0.22, p < 0.001), which in turn predicted higher cannabis frequency (β = 0.71, p < 0.001). However, neither current cannabis use (β = 0.01, p = 0.543) nor cannabis frequency (β = 0.02, p = 0.085) significantly predicted paranoia. Mediation analyses showed no significant indirect effects of trauma on paranoia via cannabis use or frequency, suggesting that trauma directly contributes to paranoia rather than through cannabis consumption. The model fit was excellent (CFI = 1.000, RMSEA = 0.036, SRMR = 0.053).


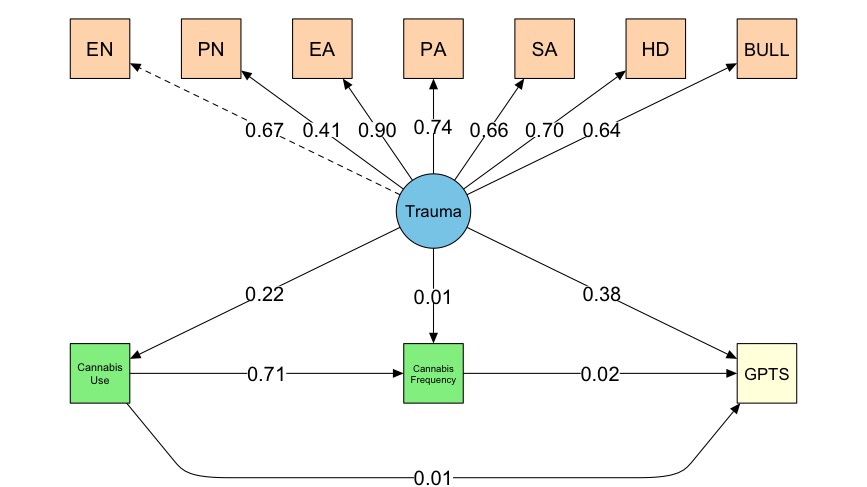


Figure S5. Structural Equation Model – Traditional Cannabis Measures.

This structural equation model (SEM) illustrates the relationships between trauma (latent variable defined by seven indicators: Emotional Neglect [EN], Physical Neglect [PN], Emotional Abuse [EA], Physical Abuse [PA], Sexual Abuse [SA], Household Discord [HD], and Bullying [BULL]), cannabis use (measured as current cannabis use and frequency of use), and Paranoia (GPTS). Standardized coefficients are displayed along the pathways, with dashed lines indicating non-significant paths.

4.5. Comparing THC Unit Analyses with Traditional Cannabis Use Measures

The use of THC units as a comprehensive measure of cannabis exposure provided critical insights beyond traditional self-reported measures, such as current use and frequency. While both approaches confirmed significant associations between cannabis use and paranoia, THC units offered a more precise dose-response relationship. Notably, in the SEM model, cannabis exposure—measured in THC units—significantly predicted paranoia, whereas traditional measures (i.e., current use and frequency) showed non-significant effects. This suggests that directly measured THC consumption, rather than frequency of use alone, is a key factor in understanding cannabis-related paranoia risk. Additionally, the THC unit model identified a small but significant indirect effect of trauma on paranoia via cannabis use, an effect not observed with traditional measures. These findings highlight the added value of THC units in psychiatric research. While frequency-based measures remain useful for broad epidemiological insights, they lack the precision for fully characterising the relationship between cannabis use and health outcomes. For example, they can help differentiate the level of risk related to consuming a smaller amount of cannabis every day (e.g., 1 unit per day) versus a larger amount of cannabis every day (e.g., 50 units per day); these would be classified as the same (i.e. daily use) when categorised by frequency of use alone. Integrating THC units into future research and clinical assessments could enhance risk detection and intervention strategies, particularly for individuals with trauma histories who are prone to paranoia.

5. Stratified Analyses by Gender

To explore potential sex differences in the associations between childhood trauma, cannabis use, and paranoia, we conducted stratified analyses for male and female participants.

Among males, nearly all trauma subtypes were significantly associated with elevated paranoia scores. The strongest effects were observed for emotional abuse (β = 11.94, p < 0.001), physical abuse (β = 9.34, p < 0.001), and bullying (β = 6.66, p < 0.001). Smaller but significant associations were found for sexual abuse, physical neglect, and household discord. Cannabis use, measured via THC units, was also a significant predictor of paranoia in males (β = 0.0063, p < 0.001). A significant household discord x THC units interaction emerged (β = 0.0082, p < 0.034).

In females, significant main effects were more selective. Paranoia was significantly elevated in relation to emotional abuse (β = 9.89, p < 0.001), bullying (β = 9.00, p < 0.001), and physical neglect (β = 4.89, p < 0.001). THC units also significantly predicted paranoia (β = 0.0085, p < 0.001), with a stronger effect size than observed in males. Notably, a significant trauma x THC units interaction was identified: a negative interaction between THC units and physical abuse (β = -0.0354, p <0 .001), suggesting a potential dampening effect.

Figures S6A and S6B display the SEM estimated separately for males and females, examining the relationships between childhood trauma, THC units, and paranoia. Among males (Figure S6A), trauma showed a strong direct association with paranoia and a smaller, but meaningfull, association with cannabis use. THC unit had a modest direct effect on paranoia, and the model explained substantial variance in both outcomes. In females (Figure S6B), similar pathways emerged, though the trauma-paranoia link was slightly stronger and the trauma-THC pathway slightly weaker. Notably, the direct effect of THC unit on paranoia was slightly higher than in males, suggestiong a potentially greater sensitivity to cannabis-related effects on paranoid thinking in females.

Overall, while trauma emerged as a robust predictor of paranoia across genders, the specific trauma subtypes and interaction patterns differed across genders. Males showed broader trauma sensitivity, whereas females demonstrated heightened reactivity to cannabis.


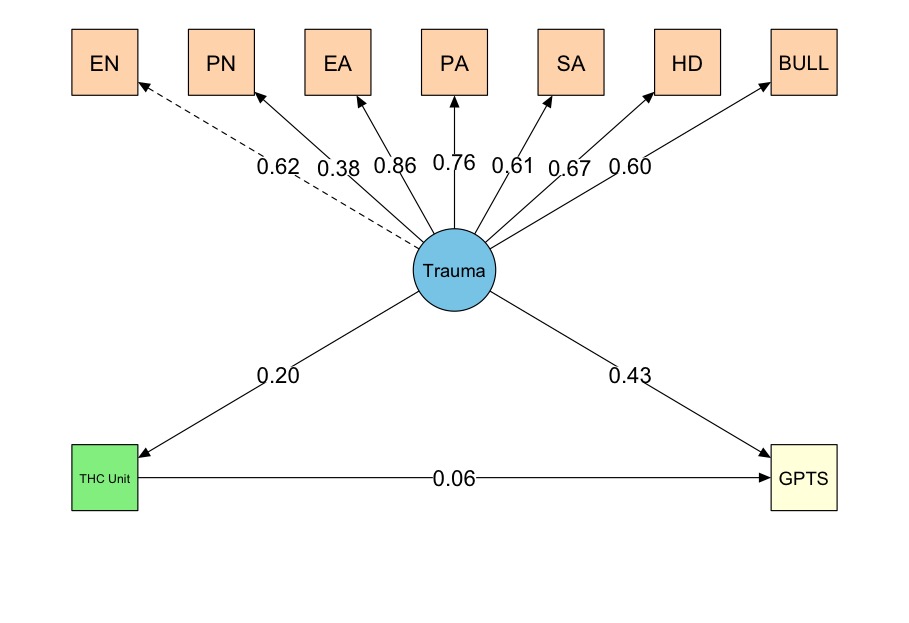


Figure S6A. Structural Equation Model – Males.

This structural equation model (SEM) illustrates the relationships between trauma (latent variable defined by seven indicators: Emotional Neglect [EN], Physical Neglect [PN], Emotional Abuse [EA], Physical Abuse [PA], Sexual Abuse [SA], Household Discord [HD], and Bullying [BULL]), cannabis use (measured as THC units), and Paranoia (GPTS). Standardized coefficients are displayed along the pathways, with dashed lines indicating non-significant paths.


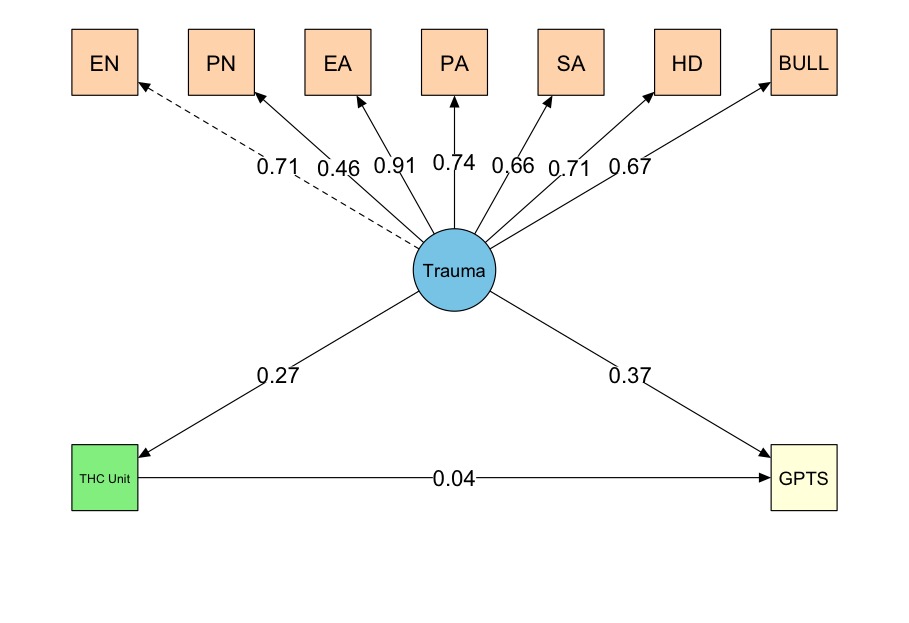


Figure S6B. Structural Equation Model – Females.

This structural equation model (SEM) illustrates the relationships between trauma (latent variable defined by seven indicators: Emotional Neglect [EN], Physical Neglect [PN], Emotional Abuse [EA], Physical Abuse [PA], Sexual Abuse [SA], Household Discord [HD], and Bullying [BULL]), cannabis use (measured as THC units), and Paranoia (GPTS). Standardized coefficients are displayed along the pathways, with dashed lines indicating non-significant paths.

6. Sensitivity Analyses Using Complete Cases

To verify the robustness of the main findings from the imputed models, we repeated the SEM analysis on complete cases (N = 3,879) (Figure S7). The model fit was acceptable (CFI = 0.990, TLI = 0.986, RMSEA = 0.078, SRMR = 0.101). In this model, trauma significantly predicted both increased cannabis use (β = 0.254, p < 0.001) and elevated paranoia scores (β = 0.397, p < 0.001). Cannabis use was negatively associated with paranoia (β = -0.040, p < 0.001). However, the indirect effect of trauma on paranoia via cannabis use, although previously significant in the imputed model, was small and non-significant in the complete-case bootstrap model (β = 0.002, SE = 0.440, p = 0.422). These findings suggest that the indirect pathway may not be robust and likely reflect alterations in the distribution of THC units and reduced statistical power due to the exclusion of missing values, particularly past cannabis users, among whom THC unit information was more often missing than among current users.


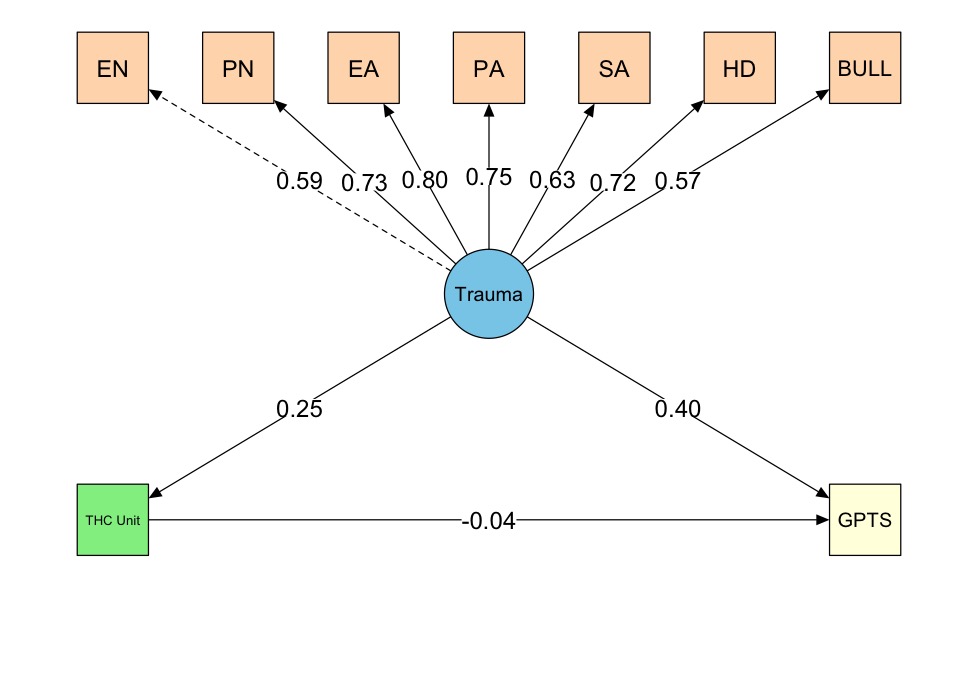


Figure S7. Structural Equation Model – Complete Cases.

This structural equation model (SEM) illustrates the relationships between trauma (latent variable defined by seven indicators: Emotional Neglect [EN], Physical Neglect [PN], Emotional Abuse [EA], Physical Abuse [PA], Sexual Abuse [SA], Household Discord [HD], and Bullying [BULL]), cannabis use (measured as THC units), and Paranoia (GPTS). Standardized coefficients are displayed along the pathways, with dashed lines indicating non-significant paths.

7. Clinical Thresholds of Paranoia

7.1. Defining Paranoia Severity Categories

To classify participants into distinct paranoia severity groups, we used the 15^th^ and 85^th^ percentiles of the Green Paranoid Thoughts Scale (GPTS) total score from our sample distribution as cut-off points. This approach allows for a meaningful differentiation between individuals with low, moderate, and high levels of paranoid thinking. As shown in Figure S8, the 15 ^th^ percentile (GPTS total score = 33) was used to define the low paranoia group, while the 85^th^ percentile (GPTS total score = 71) marked the threshold for the high paranoia group. Participants with scores falling between these two thresholds were classified as having moderate paranoia.


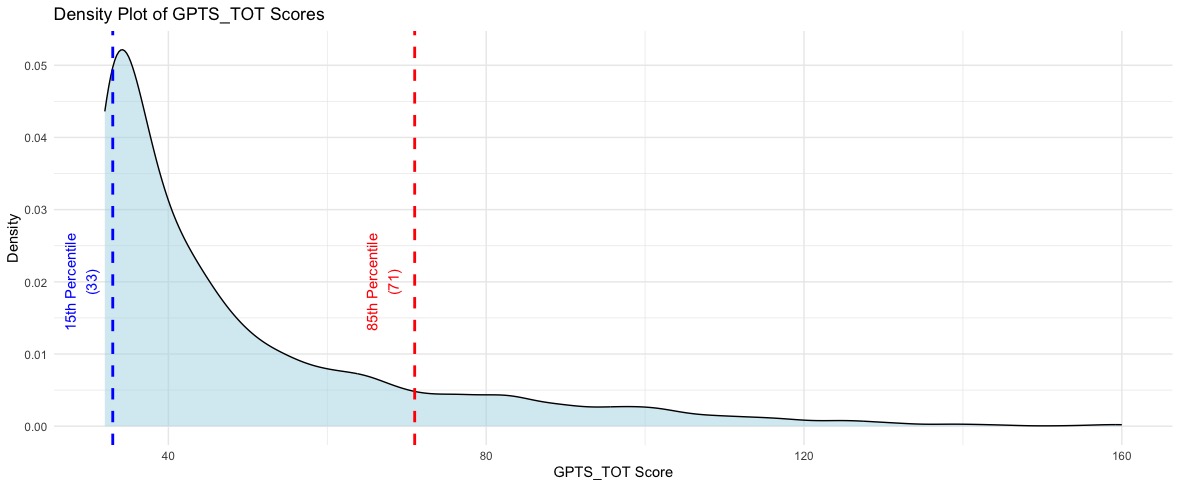


Figure S8. Density plot of GPTS total scores with Clinical Thresholds.

This figure displays the density distribution of paranoia severity (GPTS) scores across the sample. The 15^th^ percentile (GPTS total score < 33) is marked in blue, representing individuals with low paranoia, while the 85^th^ percentile (GPTS total score > 71) is marked in red, indicating high paranoia.

7.2. Paranoia Severity Across Trauma Exposure

Figure S9 highlights notable differences in paranoia severity across trauma exposure groups. Individuals who reported childhood trauma were more likely to experience high paranoia, whereas those without trauma exposure predominantly reported low or moderate levels of paranoia.

These findings align with growing evidence that childhood trauma is strongly associated with heightened paranoia, with trauma-exposed individuals experiencing more severe paranoid thoughts. This stratification approach provides a clinically relevant framework for understanding how trauma influences paranoia severity and emphasises the need for targeted interventions to mitigate paranoia in trauma-affected populations.


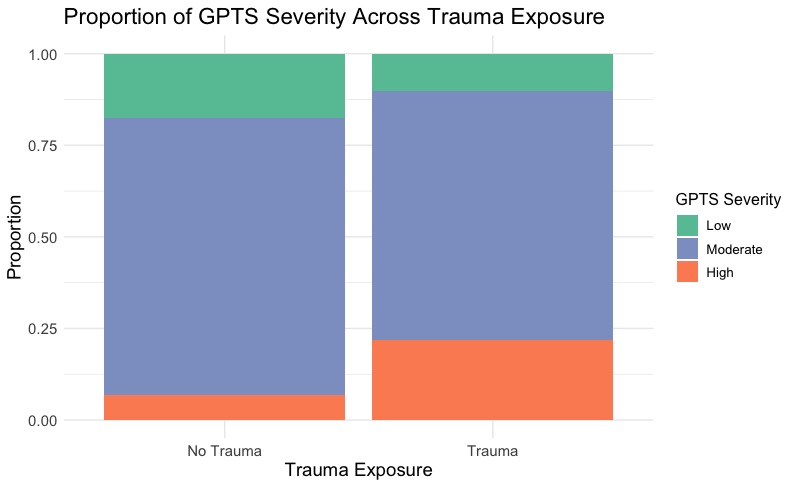


Figure S9. Proportion of Paranoia Severity by Trauma Exposure.

This figure illustrates the distribution of paranoia severity (GPTS) among individuals with and without childhood trauma exposure. Paranoia severity is categorised as follows: low (GPTS < 33), moderate (GPTS 33–71), and high (GPTS > 71).

7.3. THC Unit Distribution by Paranoia Category

**Figure S10** displays the distribution of THC unit consumption across the three paranoia categories (i.e., low, moderate, and high paranoia). Descriptive statistics indicate that individuals in the high paranoia group had the highest mean THC unit consumption (M = 183, SD = 267) compared to the moderate (M = 114, SD = 207) and low paranoia groups (M = 105, SD = 227). A Kruskal-Wallis test revealed a significant difference in THC unit consumption between paranoia groups (χ² = 19, p < 0.0001), suggesting a non-normally distributed relationship. Post-hoc Dunn’s pairwise comparisons with multiple testing correction confirmed that individuals with high paranoia reported significantly greater THC consumption than those with moderate paranoia (p < 0.0001) and those with low paranoia (p = 0.003). However, no significant difference was observed between the low and moderate paranoia groups. These findings suggest that individuals with high paranoia tend to consume more THC overall and may represent a distinct subgroup in terms of cannabis use patterns.


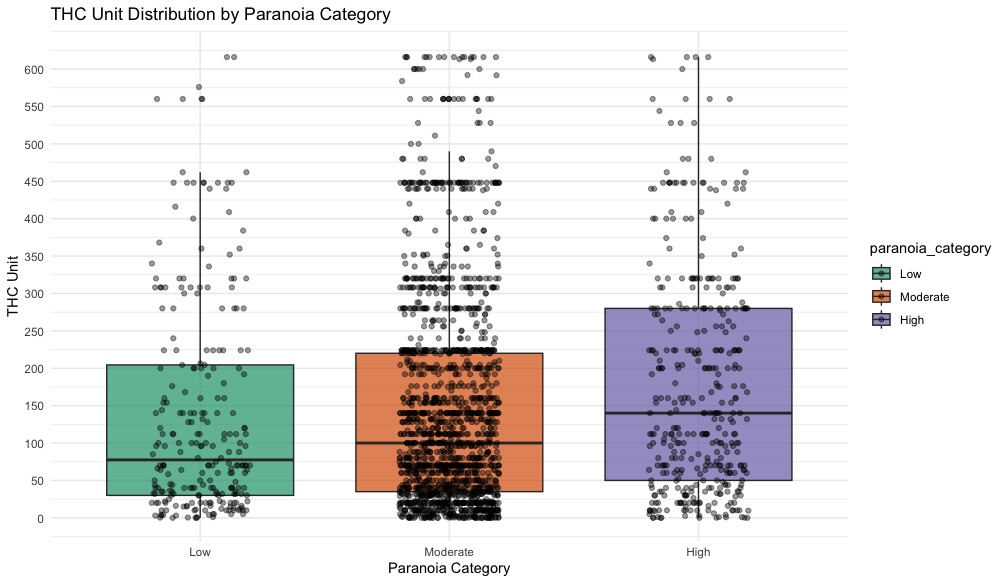


Figure S10. THC Unit Distribution Across Paranoia Categories.

This figure illustrates the distribution of THC unit consumption across the three paranoia categories. Paranoia severity is categorised as follows: low (GPTS < 33), moderate (GPTS 33–71), and high (GPTS > 71). Each box represents the interquantile range (IQR), with the horizontal line inside the box indicating the median THC unit consumption. Indvidual data points represent participant-level THC unit values.

7.4. THC by Paranoia and Trauma Exposure

**Figure S11** presents the distribution of THC unit consumption across paranoia groups further stratified by trauma exposure (trauma vs. non-trauma). Descriptive statistics indicate that within each paranoia category, individuals who reported trauma exposure generally showed higher mean THC unit consumption compared to those without trauma. Specifically, among individuals with low paranoia, those with trauma exposure consumed significantly more THC (M = 136, SD = 282) than those without trauma (M = 85, SD = 181). Similarly, within the moderate paranoia group, individuals with trauma exposure exhibited a higher THC consumption (M = 130, SD = 220) than non-trauma individuals (M = 98, SD = 192). In the high paranoia group, THC consumption remained elevated in both trauma (M = 194, SD = 273) and non-trauma (M = 146, SD = 241) subgroups, with no notable difference. A Kruskal-Wallis test was performed separately for trauma-exposed and non-trauma groups. Findings suggested that within the trauma-exposed group, THC unit consumption differed significantly across paranoia levels, with post-hoc Dunn’s pairwise tests indicating significantly higher THC use in high paranoia compared to moderate paranoia (p < 0.0001) and low paranoia (p = 0.003). No significant differences were found in the non-trauma group. As shown in **Figure S11**, individuals with both high paranoia and trauma exposure tend to consume more THC, supporting the hypothesis that early-life adversity may influence patterns of cannabis use among individuals experiencing paranoia.


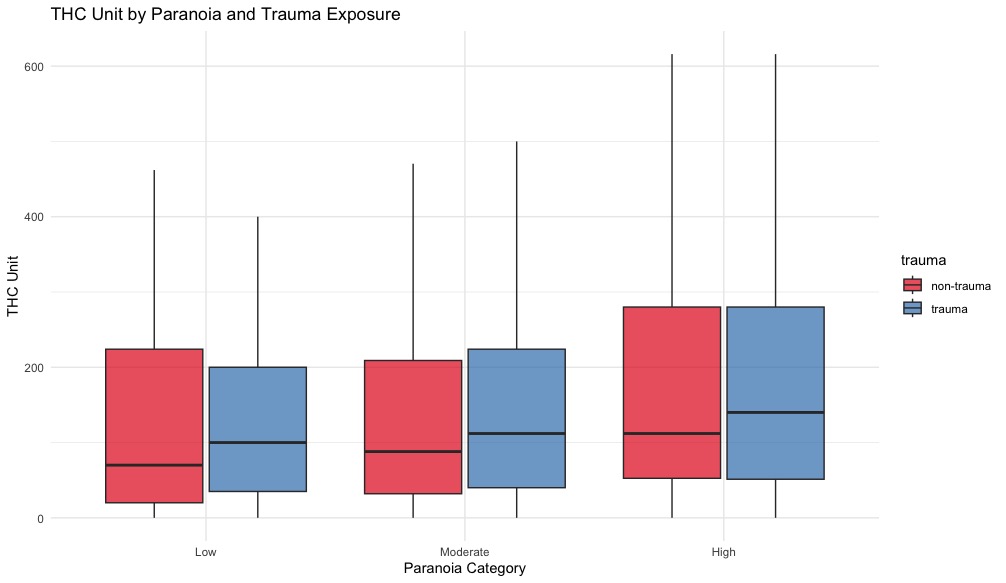


Figure S11. THC Unit Consumption by Paranoia Levels and Trauma Exposure.

This figure displays THC unit consumption stratified by both paranoia category and trauma exposure. Paranoia severity is categorised as follows: low (GPTS < 33), moderate (GPTS 33–71), and high (GPTS > 71). Red boxes represent individuals without reported trauma exposure, blue boxes represent individuals with reported trauma exposure.

7.5. Bridging General Population Findings to Clinical Relevance

While the CAMe sample is drawn from the general population, the findings of this study have important clinical implications for early detection and intervention in individuals at higher risk of developing paranoia. Even at subclinical levels, paranoia is associated with distress, social withdrawal, and functional impairment. Certain subgroups within the general population, particularly those exposed to childhood trauma and cannabis, may benefit from targeted public health strategies. Screening for trauma exposure and paranoia in non-clinical settings, such as community mental health initiatives or digital mental health platforms, could facilitate early risk identification. Additionally, public health campaigns should address the potential impact of harmful cannabis use patterns on paranoia, particularly among individuals with a history of childhood trauma. Educating the public about the risks associated with high-potency cannabis may help reduce paranoia-related distress and lower the likelihood of progression to more severe psychopathology.
